# Pulmonary cryptococcosis after recovery from COVID-19 in an immunocompetent patient: A rare case report

**DOI:** 10.1097/MD.0000000000030143

**Published:** 2022-08-12

**Authors:** Hye Sook Choi

**Affiliations:** a Department of Internal Medicine, Kyung Hee Unversity Medical Center, Seoul, Republic of Korea.

**Keywords:** COVID-19, *Cryptococcus neoformans*, immunocompetent, pulmonary cryptococcosis, SARS-CoV-2

## Abstract

**Rationale:**

*Cryptococcus neoformans* (*C neoformans*) infection typically occurs in immunocompromised patients infected with human immunodeficiency virus (HIV), or those taking immunosuppressive drugs, corticosteroids, or chemotherapy. Recently, there have been an increasing number of reports of cryptococcosis as opportunistic infections in COVID-19 patients, all of which have been related to immunocompromising conditions, underlying medical diseases, immune suppression drugs, or corticosteroids. Here, we report the first case of pulmonary cryptococcosis in an immunocompetent patient with a history of COVID-19 who had no history of underlying diseases or immune modulation drugs.

**Patient concerns:**

A previously healthy 46-year-old man presented with tiny lung nodules. He had quit smoking 6 years prior. He had no significant medical history except for COVID-19 3 months prior, and had not received corticosteroids or cytokine blockers when he had COVID-19. He had been coughing since he recovered from COVID-19.

**Diagnosis:**

Bronchoalveolar lavage cultures showed the growth of *C neoformans*. A CT-guided percutaneous needle biopsy of the lung lesion was performed. Histopathology of the biopsy specimen showed granulomas with encapsulated yeast. There was no growth of *C neoformans* in the CSF or blood. He was diagnosed with pulmonary cryptococcosis.

**Intervention:**

Antifungal drug (fluconazole) was administered for 6 months in the outside clinic.

**Outcomes:**

The lung lesions disappeared after 6 months medication.

**Lessons:**

This case may illustrate the risk of pulmonary cryptococcosis after SARS-CoV-2 infection in an immunocompetent patient. Opportunistic infections can occur even after recovery from COVID-19 for several reasons. First, SARS-CoV-2 infection causes immune dysregulation including lymphocytopenia. Second, T lymphocytes play a principal role against *Cryptococcus.* Third, these changes in the immune system due to COVID-19 may last for several weeks. Thus, we suggest careful consideration of lung lesions in patients with a history of COVID-19.

## 1. Introduction

*Cryptococcus neoformans* (*C neoformans*) is an encapsulated pathogenic yeast found in the environment that is not normally a component of the human microbial flora. *C neoformans* infections (cryptococcosis) are rare in healthy individuals, even though they inhale *C neoformans*. Cryptococcosis generally occurs in immunocompromised patients, such as those with HIV/AIDS, organ transplant, liver cirrhosis, chronic kidney disease, malignancies, diabetes, autoimmune diseases, or immunological deficiencies such as CD4 + T-cell lymphocytopenia or anti-GMCSF antibodies, or those who have been treated with immunosuppressants, corticosteroids, cytokine blockers, or chemotherapy.^[1–5]^

Recently, cases of cryptococcosis have been reported in patients with SARS-CoV-2 infection, presenting as meningoencephalitis,^[6–9]^ pneumonia,^[10,11]^ or cryptococcemia.^[12–15]^ However, all reported cases of cryptococcosis in COVID-19 patients occurred currently with an immunocompromised status, corticosteroid use, cytokine blocker use, immunosuppressants use, HIV, malignancies, or medical comorbidities.^[6–17]^ Thus far, there have been no reports of cryptococcosis in immunocompetent COVID-19 patients who had no underlying disease and no history of corticosteroid therapy.

In this case report, we describe pulmonary cryptococcosis developed in an immunocompetent patient after the acute convalescence period from COVID-19. To our best knowledge, this is the first case report of cryptococcosis related to COVID-19 who had not received any immunosuppressants or corticosteroids and had no underlying disease. We also provide a review of the related literatures.

## 2. Case presentation

**Figure 1.**
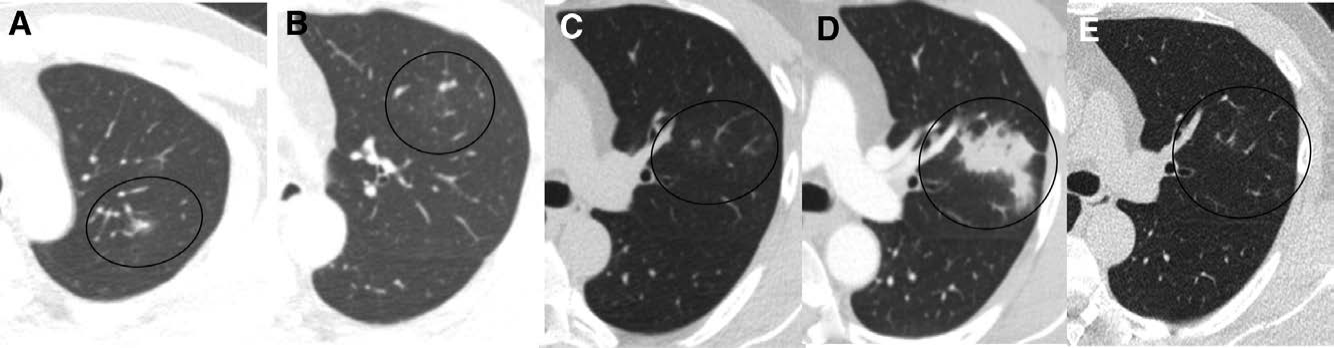
Chest CT. (A–C) Initial scattered small nodules in the left upper lobe. (D) Increased mass-like consolidations after the first bronchoalveolar lavage. (E) Disappeared mass after 6 months oral antifungal drug administration. CT = computed tomography.

A 46-year-old man was referred to our hospital from a health checkup center in July 2021 for tiny scattered nodules on a chest low-dose computed tomography (CT) scan (Fig. 1A–C). His only complaint was a dry cough, and he did not experience fever, chills, sputum, weight loss, sweating, myalgia, or fatigue. He was immunocompetent, without any underlying disease or medications. Physical examination results were normal. However, he had a history of confirmed SARS-CoV-2 infection 3 months prior to presentation. During COVID-19, he only experienced a sore throat, and his chest radiographic findings were normal. He had not received any medications for COVID-19, including corticosteroids, nonsteroidal anti-inflammatory drugs, antibiotics, cytokine blockers, or antiviral agents. After 14 days of quarantine, the patient completely recovered from COVID-19 without complications, except for an intermittent dry cough.

**Figure 2.**
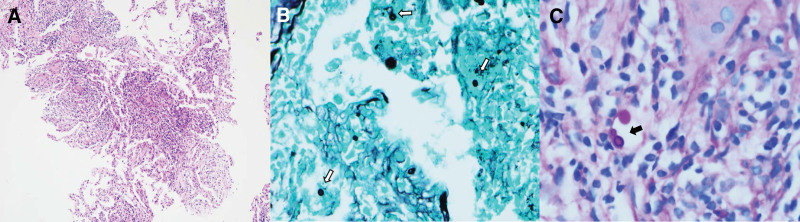
Histopathologic findings. (A) Granulomatous inflammation with multinucleated giant cells and fibrosis was observed (hematoxylin and eosin, ×100). (B) Grocott methenamine silver staining showed a few variable sized fungi with diameters ranged from 4 to 7 μm in macrophages and multinuclear giant cells (white arrows, ×400). (C) Periodic acid-Schiff staining revealed encapsulation and narrow-necked budding (black arrow, ×600) in the vessels.

His white blood cell count was 6.430/μL (50.5% segmented neutrophils, and 37.7% lymphocytes), C-reactive protein was <0.5 mg/dL, and rheumatoid factor was normal. Screening for antibodies against HIV was negative. Chest CT (Fig. 1A–C) scan from health checkup center showing scattered small nodular infiltrations on the left upper lobe (LUL) suggested bronchiolitis or tuberculosis. Thus, we performed bronchoalveolar lavage (BAL) to exclude pulmonary tuberculosis at the outpatient department. Fourteen days later, he came to the outpatient clinic to find the results. A direct smear of the BAL fluid was suspected of *C neoformans*, and the culture results were pending. And follow-up chest X-ray showed progression of the LUL lesions. However, he did not complain about any additional symptoms except dry cough. The patient was hospitalized to evaluate for invasive cryptococcosis. We performed contrast-enhanced chest CT (Fig. 1D) to evaluate the increased consolidative mass on the chest X-ray. The second BAL and percutaneous needle biopsy (PCNB) of the left upper lobe mass (Fig. 1D) were performed to exclude other microorganisms. *C neoformans* was identified in the culture medium of the second BAL fluid. Antigen testing for *Cryptococcus* was positive. Histological findings from PCNB revealed granulomatous inflammation with encapsulated rounded yeast form fungi in macrophages and multinuclear giant cells. These findings demonstrated a *C neoformans* infection (Fig. 2A–C). We performed cerebrospinal fluid (CSF) tapping and blood culture to exclude disseminated cryptococcosis. There was no growth of *C neoformans* in the CSF or blood. The echocardiographic findings were normal. We confirmed pulmonary cryptococcosis after COVID-19 recovery in an immunocompetent patient. He was treated with an antifungal agent (fluconazole 400 mg/day) for lung lesions. The patient had no adverse events of therapeutic drug. After 6 months of treatment, the consolidative cryptococcosis masses disappeared (Fig. 1E) without any complications.

## 3. Discussion and Conclusions

Immunocompromised conditions are major risk factors for cryptococcosis.^[1–5]^ Recently, *C neoformans* infections have been increasingly reported in COVID-19 patients.^[6–11]^ However, all prior cases have been linked to immunocompromised conditions caused by either an underlying disease, immunodeficiency, or immunosuppressive drugs such as corticosteroids, cytokine blockers, or chemotherapy.^[6–15]^ Although there are cases of cryptococcosis that have reported in immunocompetent patients related to COVID-19, they were also patients who had hypertension,^[10,16,17]^ diabetes,^[10,17]^ chronic kidney disease,^[16]^ cirrhosis,^[16]^ or treated with corticosteroid for COVID-19.^[9]^ In case of pulmonary cryptococcosis developed 2 months after recovering from COVID-19, also the patient had hypertension, diabetes, and treatment with corticosteroids for COVID-19.^[10]^ The patient in the present case had not received any immunosuppressive drugs or corticosteroids for COVID-19 and had no medical comorbidities or immunodeficiency diseases. Thus, this is the first report of cryptococcosis diagnosed in an immunocompetent patient who had COVID-19 3 months previously.

The initial tiny nodules on the chest CT scan conducted at the health checkup center progressed to a consolidative mass 14 days after BAL. No microorganisms other than *Cryptococcus* were detected from the PCNB and culture. It was assumed that the BAL might worsen the cryptococcal seeding in the patient.

Generally, SARS-CoV-2 causes hyperinflammation and cytokine storm syndrome. Immunosuppressive drugs such as corticosteroids or cytokine blockers have been administered to alleviate these unexpected inflammatory responses in patients with COVID-19. Unfortunately, these immunosuppressive therapies for COVID-19 increase opportunistic infections.^[15]^

The current patient developed pulmonary cryptococcosis after recovering from COVID-19. This suggests that changes in immune regulation following SARS-CoV-2 infection persist even after recovery from COVID-19. Even in people who have fully recovered from COVID-19, changes in the immune system caused by SARS-CoV-2 infection raise concerns about opportunistic infections. This is mainly, because the immune system does not fully recover even though the symptoms fully disappear after COVID-19.

Although the exact mechanism by which COVID-19 induces or promotes fungal infection has not been identified, many reports have suggested that SARS-CoV-2 infection is the predisposing factor of fungal infection.^[18–20]^ SARS-CoV-2 infection promotes pulmonary microbial proliferation and alters the human microbiota, which may affect the immune system and contribute to fungal infections.^[18]^ SARS-CoV-2 infection is associated with immune dysregulation and global T-cell depletion.^[19]^ COVID-19 patients show increased circulating proinflammatory cytokines and significantly decreased absolute numbers of T lymphocytes, CD4 + T cells, and CD8 + T cells.^[20–23]^ These immune system alterations after SARS-CoV-2 infection may increase susceptibility to fungal infections.^[22]^ Lymphocytes are an important component of the host defense against *Cryptococcus*. IL-2-activated T cells and NK cells form conjugates with *C neoformans* and directly inhibit the growth of *Cryptococcus.*^[24]^ T cells (CD4 + and CD8+) and NK cells (CD16/56+) have the capacity to bind and directly inhibit the growth of *C neoformans.*^[25]^ These immune modulations from SARS-CoV-2 infection might affect the protective immune response against fungal infections. The resolution of lymphopenia correlates with recovery from COVID- 19, but this may take several weeks.^[26]^ This case highlights that the changes in the immune system occurring due to SARS-CoV-2 infection in immunocompetent patients may persist even after convalescence from the initial illness.

This study has several limitations. First, we did not measure the absolute numbers of T lymphocytes and NK cells in the patient. Thus, we could not clearly identify the pathogenesis of cryptococcosis. Second, although the patient did not have any underlying disease at the time of diagnosis, the possibility of an incubation period for immunodeficiency disease cannot be completely ruled out. However, there are currently no test methods to identify this.

SARS-CoV-2 infection has been conclusively shown to activate the innate and adaptive immune responses, which decreases the number of T-cell lymphocytes and NK T cells, which play a critical role in host defense against *Cryptococcus*. We hypothesized that the changes in the immune profile from SARS-CoV-2 infection might be sustained for several weeks even though it was eradicated, which can cause fungal infection.

## Author contributions

All authors participated in the management of the patient described in this case report. HSC collected all the references and was a major contributor in the writing of the article. All authors have read and approved the article.

Conceptualization: Hye Sook Choi

Data curation: Hye Sook Choi

Formal analysis: Hye Sook Choi

Investigation: Hye Sook Choi

Methodology: Hye Sook Choi

Resources: Hye Sook Choi

Supervision: Hye Sook Choi

Validation: Hye Sook Choi

Writing—original draft: Hye Sook Choi

Writing—review and editing: Hye Sook Choi
